# An Amazonian *Ganoderma* isolate as a source of antioxidant proteins: Influence of submerged fermentation conditions on mycelial architecture and bioactivity

**DOI:** 10.1007/s42770-026-01934-8

**Published:** 2026-04-22

**Authors:** Vítor Alves Pessoa, Giovanna Lima-Silva, Aldenora dos Santos Vasconcelos, Lorena Vieira Bentolila de Aguiar, Enedina Nogueira de Assunção, Walter José Martinez-Burgos, Ceci Sales-Campos, Larissa Ramos Chevreuil

**Affiliations:** 1 Postgraduate Program in Biotechnology, Universidade Federal do Amazonas, Av. General Rodrigo Octavio, Amazonas, 69067-005 Brazil; 2 Edible Fungi Cultivation Laboratory, Instituto Nacional de Pesquisas da Amazônia, Av. André Araújo, Amazonas, 69067-375 Brazil; 3 Postgraduate Program in Biodiversity and Biotechnology of the Bionorte Network, Universidade do Estado do Amazonas, Av. Carvalho Leal, Amazonas, 69065-001 Brazil; 4 Multiuser Center for the Analysis of Biomedical Phenomena, Universidade do Estado do Amazonas, Av. Carvalho Leal, Amazonas, 69065-001 Brazil; 5 Multidisciplinary Support Center, Instituto de Ciências Biológicas, Universidade Federal do Amazonas, Av. General Rodrigo Octavio, Manaus, Amazonas 69067-005 Brazil

**Keywords:** Amazonian *Ganoderma*, Mycelial biomass, Submerged fermentation, Antioxidant activity, Protein extracts

## Abstract

*Ganoderma* sp. 1962 is an Amazonian isolate with reported potential for the production of bioactive compounds; however, the effects of specific submerged fermentation parameters on mycelial architecture and on the recovery and bioactivity of protein extracts remain poorly understood. In this study, the influence of different submerged fermentation conditions on the recovery of antioxidant proteins from *Ganoderma* sp. 1962 was evaluated. Molecular identification was performed by sequencing the internal transcribed spacer (ITS) region followed by phylogenetic analysis. The isolate was cultivated under four nutrient compositions combined with agitated (A) and non-agitated (NA) regimes, resulting in eight experimental conditions. Mycelial biomass was structurally characterized using Scanning Electron Microscopy (SEM) and Attenuated Total Reflection–Fourier Transform Infrared (FTIR-ATR) spectroscopy. Protein extracts obtained from the mycelial biomass were analyzed for soluble protein content, total phenolic compounds (TPC), and reducing sugars (RS). Antioxidant activity was assessed using ABTS^•+^, DPPH^•^, metal chelating ability, and reducing power assays. BLAST analysis of ITS rDNA sequences revealed high similarity (> 98% identity) to *Ganoderma multiplicatum*, which was subsequently confirmed by phylogenetic analysis using reference sequences from GenBank. Biomass production ranged from 5.57 to 8.73 g L^− 1^, with marked variation in glucose consumption among conditions and a stronger tendency toward pH acidification under agitated cultivation. Macromorphological differences were predominantly characterized by pellet formation under agitated cultivation and by mycelial aggregates under static conditions. Micromorphological analysis revealed cultivation-dependent traits, including the formation of short, highly branched “staghorn” hyphae under non-agitated conditions, whereas agitated cultures promoted long, filamentous hyphae. FTIR-ATR analysis showed significant spectral differences among samples, indicating that culture medium composition strongly influenced the chemical composition of the biomass. The E3NA condition (Experiment 3, non-agitated) yielded the highest soluble protein content (218.35 ± 16.95 mg g^− 1^ extract), while E1A (Experiment 1, agitated) exhibited the highest levels of RS (25.50 ± 1.59 mg g^− 1^ extract) and TPC (31.96 ± 1.39 mg GAE g^− 1^ extract). The E1A extract showed the strongest antioxidant activity, with values of 90.34 ± 0.66% (ABTS^•+^), 68.09 ± 1.74% (DPPH^•^), and 0.335 ± 0.011 (reducing power), suggesting that protein–phenol and protein–carbohydrate interactions may contribute to antioxidant activity, as supported by correlation analysis. Although non-agitated fermentation favored soluble protein recovery, protein–metabolite interactions may play a more relevant role in antioxidant activity. Overall, this study provides novel evidence of the potential of *G. multiplicatum* to produce antioxidant proteins under submerged fermentation.

**Supplementary Information:**

The online version contains supplementary material available at 10.1007/s42770-026-01934-8.

## Introduction

The genus *Ganoderma* comprises a group of medicinally important mushroom widely recognized for their ability to produce a diverse array of bioactive compounds with pharmacological relevance, including terpenoids, polysaccharides, steroids, alkaloids, and proteins [1, 2]. Among these, polysaccharides and triterpenes have been extensively investigated, whereas proteins and other metabolite classes remain comparatively underexplored despite their significant biological potential [3].

Proteins derived from *Ganoderma* comprise diverse functional classes with recognized relevance to human health. Among these, fungal immunomodulatory proteins (FIPs) have attracted particular attention due to their immunomodulatory, antitumor, antiviral, antiallergic, hemagglutinating, and antioxidant activities, among others [4]. In this context, the Amazon region represents a particularly promising reservoir of unexplored fungal diversity, offering the potential to identify novel strains capable of synthesizing proteins with unique biochemical and functional characteristics [5].

An example of this potential is the fungus *Ganoderma* sp. 1962, isolated in Manaus, Amazonas, Brazil [6]. This strain has shown notable protein synthesis capacity, evidenced by increased protein content in post-cultivation residues [6] and the production of proteins of pharmacological interest, such as fibrinolytic enzymes and protease inhibitors, when cultivated on Amazonian lignocellulosic residues [7].

Comparative studies between *Ganoderma* sp. 1962 and a commercial strain of *Ganoderma sichuanense* revealed significant differences in mycelial biomass production during submerged fermentation [8]. However, it is important to emphasize that cultivation conditions play a key role in protein content, which is not always correlated with high mycelial biomass production [9]. This dissociation highlights the need not only to optimize cultivation strategies for protein recovery but also to employ analytical approaches capable of rapidly assessing biomass composition.

In this context, rapid and non-invasive analytical techniques have become increasingly important. Fourier Transform Infrared (FTIR) spectroscopy has proven to be an effective tool for the identification and characterization of biopolymers and other metabolites [10]. Attenuated Total Reflectance–FTIR (FTIR-ATR), in particular, enables non-destructive analysis of protein-related functional groups, making it suitable for cost-effective and scalable industrial applications [11]. However, chemical composition alone does not fully explain differences in metabolite synthesis, as fungal physiology is also strongly influenced by morphological organization under submerged cultivation.

In parallel, morphological engineering has gained prominence as an approach to correlate fungal morphology with the synthesis of specific metabolites [12]. Studies in this area demonstrate, for example, changes in cell wall structure, pellet formation, and the synthesis of selenium-enriched proteins by *Ganoderma lucidum* as mechanisms to increase tolerance to sodium selenite in submerged culture [13]. Nevertheless, most evidence has focused on metabolite classes other than proteins, and the influence of cultivation-driven morphological traits on the functional performance of protein extracts under submerged fermentation remains poorly understood.

Despite previous reports describing the protein-producing capacity of *Ganoderma* sp. 1962, the relationship between submerged fermentation conditions, mycelial architecture, and antioxidant bioactivity has not been clearly established. Therefore, this study aimed to investigate how defined submerged fermentation conditions modulate mycelial morphology, biomass composition, and the antioxidant properties of protein-rich extracts obtained from an Amazonian *Ganoderma* isolate. By integrating microscopic analysis, biochemical characterization, and antioxidant assays, this work seeks to elucidate the relationship between cultivation conditions, mycelial architecture, and antioxidant bioactivity in submerged fungal cultures.

## Materials and methods

### Biological material and molecular characterization

The macrofungus *Ganoderma* sp. isolate 1962 was provided by the Edible Fungi Cultivation Laboratory, Technology and Innovation Coordination at the Instituto Nacional de Pesquisas da Amazônia (COTEI-INPA), Manaus, Amazonas, Brazil. The isolate was maintained on Potato Dextrose Agar (PDA) for preservation and routine handling. Submerged fermentation was performed in glucose–yeast extract–peptone (GYP) medium at pH 6.0, incubated at 25 °C for 10 days under static conditions, as previously reported for *Ganoderma* spp. [9]. Static conditions were adopted to obtain standardized mycelial biomass based on preliminary cultivation tests for genomic DNA extraction, as agitated cultures frequently promote polysaccharide accumulation, which can interfere with DNA extraction efficiency and purity.

Recovered mycelial biomass was macerated in liquid nitrogen, and genomic DNA was extracted using the PureLink™ Genomic DNA Kit (Invitrogen™), following the manufacturer’s instructions. DNA integrity and extraction efficiency were assessed by 1% agarose gel electrophoresis. The Internal Transcribed Spacer (ITS) region was amplified by polymerase chain reaction (PCR) using the primer pairs ITS1/ITS4 and ITS5/NL4, and the amplicons were analyzed on 0.8% agarose gels. Sequencing was performed using the BigDye™ Terminator v3.1 kit on a 3500 Genetic Analyzer (Applied Biosystems). Sequence quality was evaluated using Phred quality scores, and forward and reverse reads were assembled into a consensus sequence. The resulting sequence was compared with reference sequences deposited in the NCBI database using BLASTn. The ITS sequence generated in this study was deposited in GenBank under the accession number PX736081.

### Phylogenetic tree construction

The phylogenetic tree was inferred using the Maximum Likelihood method in MEGA X with the Kimura 2-parameter model with a discrete Gamma distribution (+ G) to account for rate variation among sites and 1000 bootstrap replicates. Tree visualization was performed using iTOL v7. The dataset comprised ITS sequences of *Ganoderma* species from NCBI/GenBank, prioritizing type-derived or reference sequences (Online Resource 1), and was rooted using *Tomophagus colossus* as the outgroup [14–44].

### Submerged fermentation

*Ganoderma* sp. 1962, grown on PDA medium, was used as the inoculum for submerged fermentation. Seven mycelial discs (Ø = 7 mm) were inoculated into 250 mL Erlenmeyer flasks containing 125 mL of culture medium with different compositions (Table 1) at pH 6.0. The flasks were incubated for 12 days at 25 °C, either under agitation at 120 rpm (A) or non-agitated (NA) conditions, resulting in eight experimental conditions, based on cultivation parameters previously reported by our research group [9]. For each experimental condition, a total of 25 Erlenmeyer flasks were prepared to ensure sufficient biomass for downstream analyses.

**Table 1:** Composition of culture media across different experiments

Culture Media	Concentration (g L^− 1^)		
	Glucose	Soy Peptone	Yeast Extract
Experiment 1 (E1)	10	2,5	5
Experiment 2 (E2)	20	2,5	5
Experiment 3 (E3)	10	5	5
Experiment 4 (E4)	20	5	5

### Production parameters

Following the fermentation period, three flasks per condition were randomly selected and used as independent biological replicates for the determination of production parameters. The contents were centrifuged (10,000 rpm, 10 min, 20 °C) to separate the mycelial biomass from the fermented broth. Mycelial biomass production (g L^− 1^) was estimated based on dry weight after oven-drying at 60 °C. Glucose consumption was determined by quantifying reducing sugars (RS) in the fermented broths using the 3,5-dinitrosalicylic acid (DNS) method. For this purpose, three flasks containing culture medium without inoculation were used as initial controls (day 0), and glucose consumption was calculated by subtracting the RS concentration at day 12 from the initial values [9]. The remaining Erlenmeyer flasks were used for protein extraction and subsequent analyses.

### Morphological analysis

Fungal macromorphology was documented after fermentation by digital photography prior to broth separation. For micromorphology analysis, in natura mycelial biomass fragments (~ 0.5 cm) were fixed in modified Karnovsky’s solution (2% formaldehyde and 2.5% glutaraldehyde in 0.2 M sodium cacodylate buffer, pH 7.4) for 24 h at room temperature (25 °C). Samples were washed four times in sodium cacodylate buffer (0.2 M, pH 7.4) and post fixed for 2 h in a solution containing 1% osmium tetroxide and 0.8% potassium ferrocyanide (1:1, v/v) at room temperature. Samples were dehydrated through a graded ethanol series (30%, 50%, 70%, 80%, 90%, and three changes of 100%) for 20 min at each step, followed by critical point drying (Leica EM CPD300). The dried material was mounted on aluminum stubs using carbon tape and sputter coated with platinum to a thickness of approximately 10 nm using a Leica EM ACE600. Micrographs were acquired at various magnifications using a scanning electron microscope (JEOL JSM IT500HR) operated at an accelerating voltage of 5 kV in secondary electron mode. Morphological observations were qualitatively assessed and interpreted in relation to biochemical composition and antioxidant activity data.

### Infrared spectroscopy analysis

Spectra were obtained using an attenuated total reflectance-Fourier transform infrared spectrophotometer (FTIR-ATR, Cary 630) in the 4000 to 400 cm^− 1^ spectral range at a resolution of 8 scans. Briefly, 5 mg of each lyophilized and ground mycelial biomass was analyzed at room temperature (25 °C), with background spectra collected before each measurement. Spectra were obtained from pooled biomass representing each experimental condition [11].

### Protein extraction

Mycelial biomasses (5 g) were subjected to soluble protein extraction in 0.15 M sodium chloride (1:20 w/v) under agitation at 120 rpm for 2 h at 10 °C. The material was centrifuged (10,000 *xg*, 20 min, 4 °C), and the supernatant was dialyzed against distilled water for 48 h at 4 °C using 12 kDa molecular weight cut-off cellulose membranes, yielding protein fractions of medium to high molecular mass. Finally, the dialyzed material was centrifuged again (4.667 x*g*, 20 min, 4 °C), followed by lyophilization [9].

### Biomolecules quantification

Protein concentration in the extracts was estimated by the Bradford method [45], adapted for 96-well plates, using a bovine serum albumin (BSA) standard curve. Total phenolic compound (TPC) content was determined by the Folin-Ciocalteu method, with results expressed as milligrams of Gallic Acid Equivalents (GAE) per gram of sample [46]. Reducing sugars were quantified by the DNS method, with values estimated from a glucose standard curve [9]. All measurements were performed in triplicate.

### Antioxidant activity

Antioxidant activity in the protein extracts (5 mg mL^− 1^) was determined through 2,2′-azino-bis(3-ethylbenzothiazoline-6-sulfonic acid) (ABTS^•+^), 2,2-diphenyl-1-picrylhydrazyl (DPPH^•^), ferrous ion chelating capacity, and ferric reducing antioxidant power (FRAP) assays, using methodologies adapted for 96-well microplates. In the reducing power assay, increased absorbance indicated higher antioxidant potential [47]. All assays were performed in triplicate.

### Experimental design and statistical analysis

The experiments were conducted under a completely randomized design in a 4 × 2 factorial scheme (four culture media × two cultivation conditions). All quantitative analyses were performed in triplicate and subjected to analysis of variance (ANOVA). Means were compared by Tukey’s test at a 1% significance level (*p* < 0.01) using Statistica 7 software. FTIR-ATR data were processed using chemometric analysis through Principal Component Analysis (PCA), and Pearson correlation analysis between biomolecules and antioxidant activity was performed, both analyses using OriginPro 2025 software.

## Results

### Identification of the isolate

**Fig. 1:**
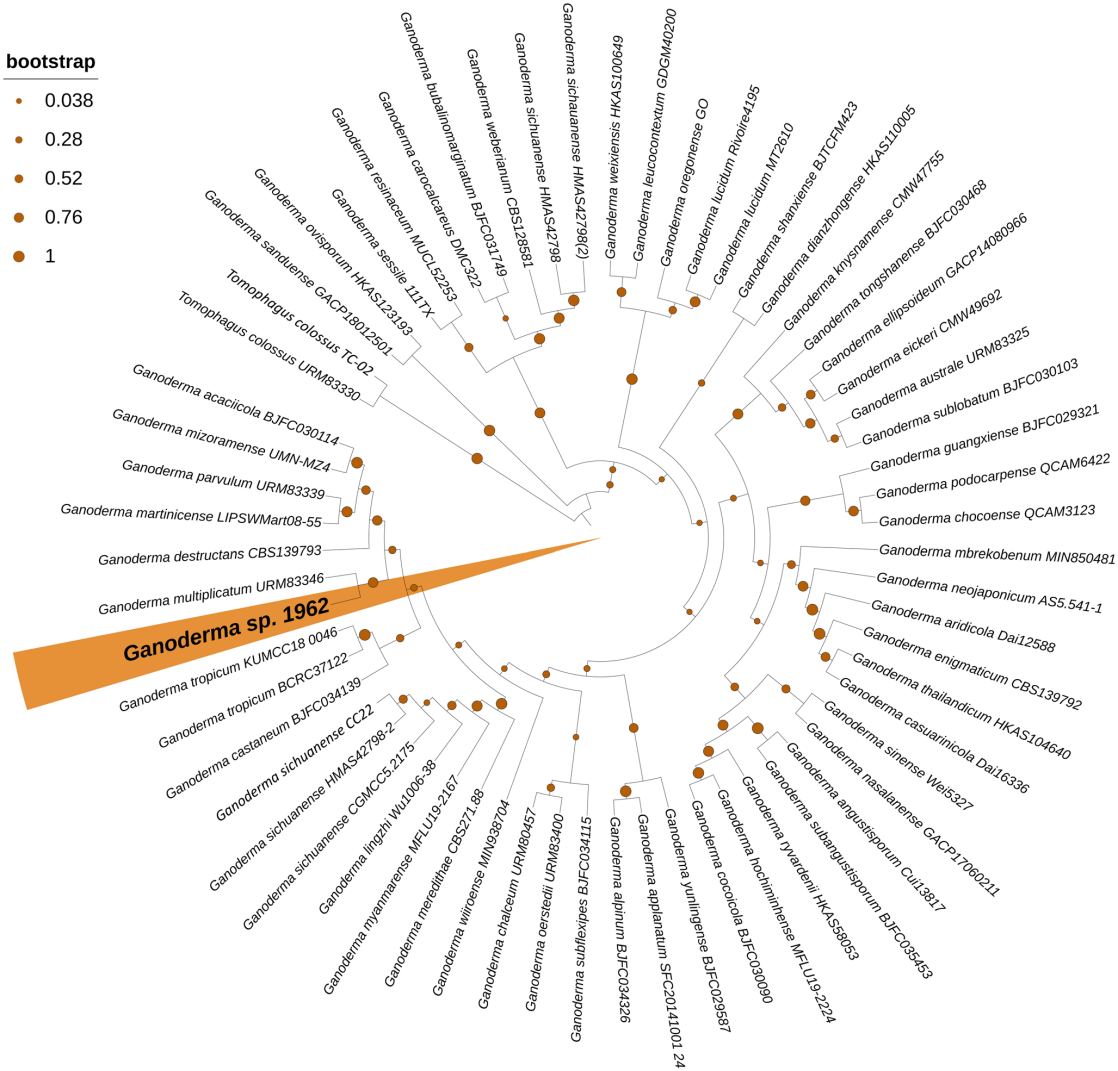
Maximum Likelihood phylogenetic tree based on ITS region sequences, illustrating the placement position of the Amazonian isolate *Ganoderma multiplicatum* 1962 (highlighted in orange). Bootstrap values (1000 replicates) are indicated at the nodes. *Tomophagus colossus* was used as the outgroup

The obtained sequence showed high similarity to fungi within the order Polyporales, particularly species of the genus *Ganoderma*, when compared with sequences deposited in the NCBI database. The highest similarities were observed with *Ganoderma* sp. strains CIRM-BRFM 1410 and 2361 (99.10% identity; 100% query coverage; E-value 0.0), as well as *G. multiplicatum* isolates CC8 (98.74% identity; 100% query coverage; E-value 0.0) and Dai 17,395 (98.73% identity; 99% query coverage; E-value 0.0). In the phylogenetic tree constructed using representative *Ganoderma* sequences, the Amazonian isolate clustered within a single clade together with *G. multiplicatum* (Fig. 1).

### Growth parameters

**Fig. 2:**
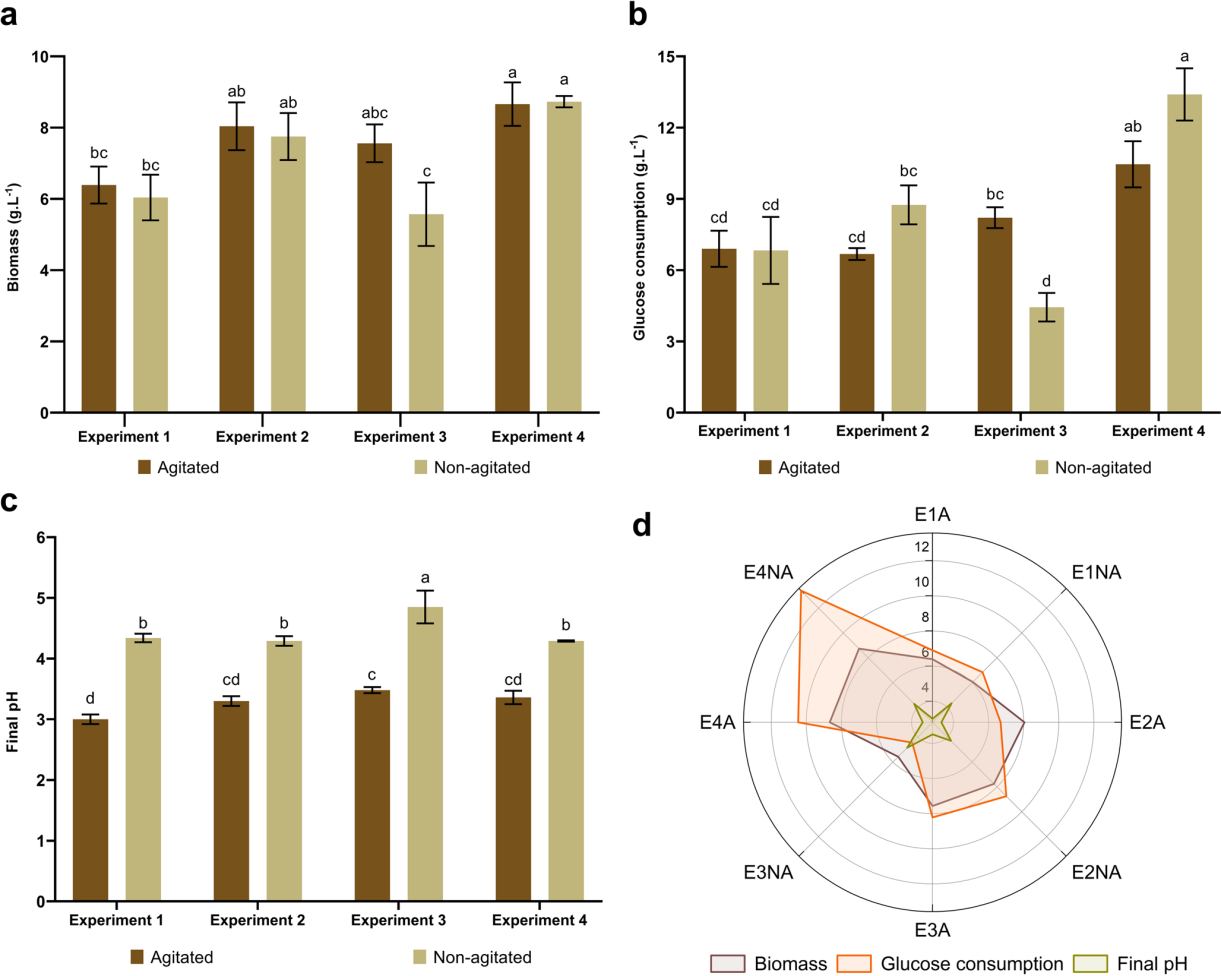
Production parameters of *Ganoderma multiplicatum* 1962 cultivated under different submerged fermentation conditions. (**a**) Dry biomass, (**b**) glucose consumption during fermentation, (**c**) final pH, and (**d**) radar chart summarizing the evaluated production parameters. Data are expressed as mean ± standard deviation (*n* = 3). Distinct letters above the bars indicate statistically significant differences between treatments (*p* < 0.05) according to one-way ANOVA followed by Tukey’s post hoc test

Cultivation of *G. multiplicatum* 1962 under different fermentation conditions resulted in mycelial biomass values ranging from 5.57 ± 0.89 g L^− 1^ (E3NA) to 8.73 ± 0.16 g L^− 1^ (E4NA) (Fig. 2a). Glucose consumption by *G. multiplicatum* varied from 4.44 g L^− 1^ (E3NA) to 13.40 g L^− 1^ (E4NA) (Fig. 2b).

Medium acidification was observed under all evaluated conditions, with lower final pH values in agitated cultures (Fig. 2c). Condition E1A showed the lowest pH (3.00 ± 0.08), while E3NA exhibited the highest pH (4.85 ± 0.27). E4NA and E4A presented the highest biomass production and glucose consumption among the evaluated conditions (Fig. 2d).

### Fungal morphology

**Fig. 3:**
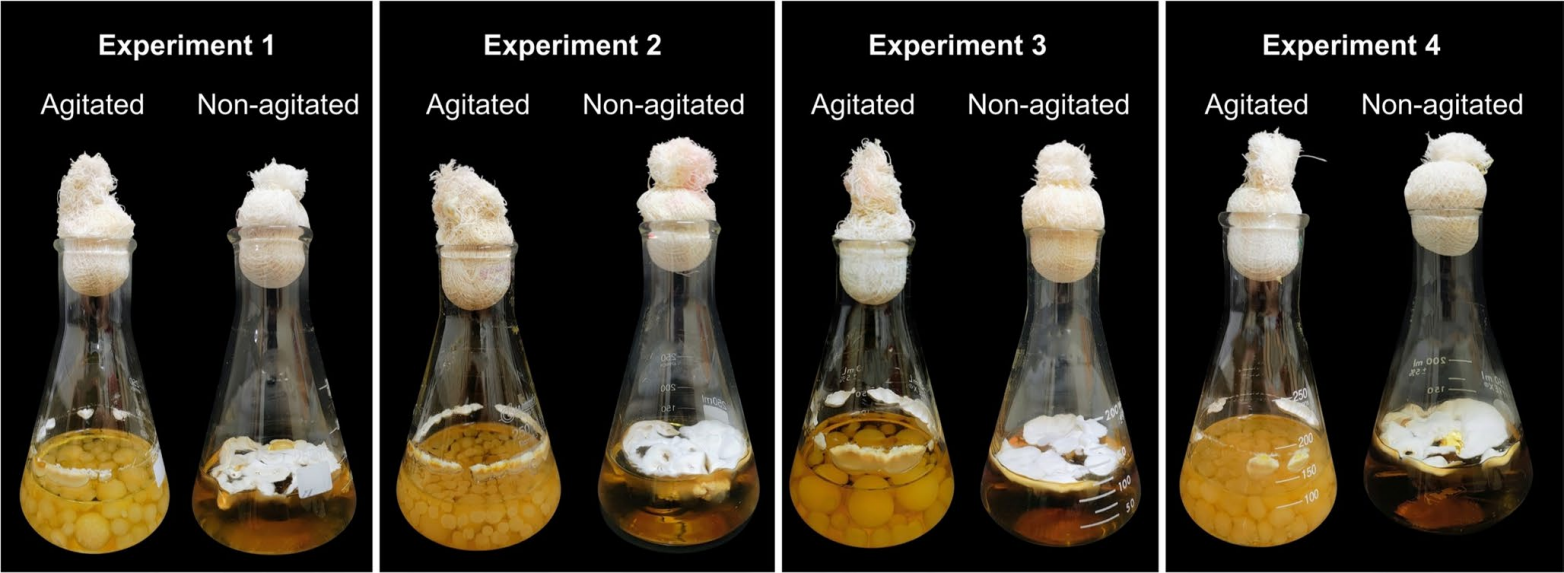
Macromorphology of *Ganoderma multiplicatum* 1962 cultivated under different submerged fermentation conditions. Representative images of mycelial growth under agitated and non-agitated conditions for Experiments 1–4

Under agitated conditions, *G. multiplicatum* exhibited pelletized growth, characterized by spherical structures with smooth surfaces. In contrast, static cultivation resulted in interwoven mycelial aggregates dispersed on the medium surface (Fig. 3).

Pellet size varied among conditions: E1A showed pellets of variable size, E2A predominantly small pellets, E3A medium-to-large pellets, and E4A medium-sized pellets (Fig. 3). Variations in culture medium coloration were also observed, including the presence of copper-toned media in some conditions. In agitated cultures, particularly in experiments 2 and 3, mycelial clusters adhered to the Erlenmeyer flask walls (Fig. 3).

**Fig. 4:**
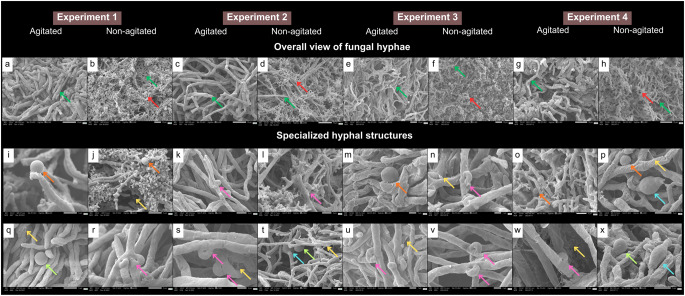
Micromorphology of *Ganoderma multiplicatum* 1962 mycelial biomass obtained under different submerged fermentation conditions. Scanning electron microscopy (SEM) images showing overall hyphal organization (**a**–**h**) and specialized hyphal structures (**i**–**x**) under agitated and non-agitated conditions across the four experimental setups. Colored arrows indicate specific morphological features: dark green, tubular hyphae; red, staghorn hyphae; orange, ornamented spherical structures; light green, smooth-surfaced spherical structures; pink, clamp connections; light blue, chlamydospores; yellow, extracellular matrix

Micromorphological differences were observed between incubation conditions. Agitated cultures exhibited long, rigid, tubular filamentous hyphae, whereas static cultures showed short and highly branched (“staghorn”) hyphae (Fig. 4). Small ornamented spherical structures deposited on the hyphae were observed in E1A, E2NA, E3A, E4A, and E4NA (Fig. 4i, j,m, o,p). Smooth-surfaced spherical structures associated with the hyphae were observed in E1A, E2NA, and E4NA (Fig. 4q, t,x). Clamp connections were observed in all experimental conditions except E1A (Fig. 4). Chlamydospores were identified in E2NA and E3NA (Fig. 4t, x). Extracellular matrix (ECM) structures with a spiderweb-like morphology covering the hyphae were observed across all cultivation conditions (Fig. 4).

### FTIR and chemometric analysis

**Fig. 5:**
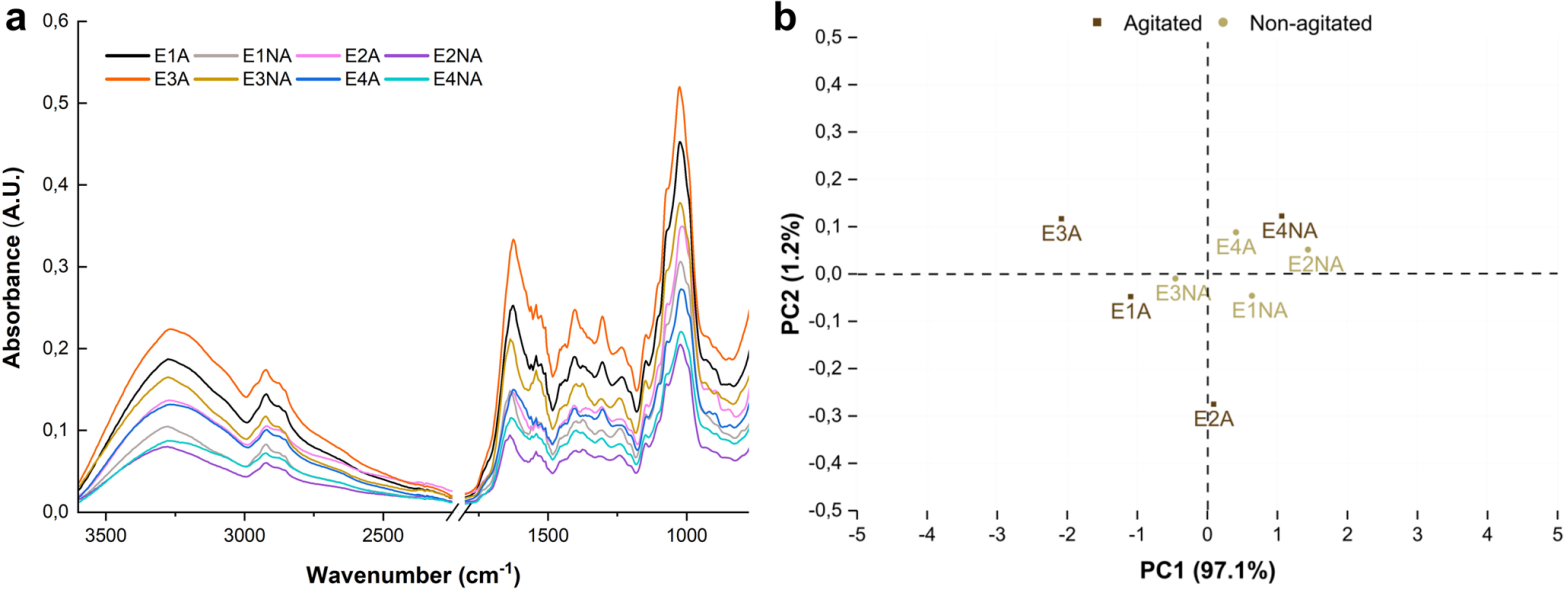
(**a**) FTIR-ATR spectra of *Ganoderma multiplicatum* 1962 mycelial biomass samples obtained under different submerged fermentation conditions and (**b**) Principal Component Analysis (PCA) score plot based on the spectrometric data. A.U.: absorbance units. A: agitated; NA: non-agitated; E: experiment (1–4)

FTIR-ATR analysis showed a consistent profile of absorption bands across all treatments, with similar spectral patterns observed among the mycelial biomasses obtained under different fermentation conditions (Fig. 5a). Differences in absorbance intensity were observed among conditions, particularly for E3A and E1A, which showed the highest absorbance values.

The spectra exhibited prominent absorption peaks at 3270 (O–H and N–H stretching), 2924 (C–H stretching), 1624 (amide I, C = O stretching), and 1544 cm^− 1^ (amide II, N–H bending), as well as bands at 1403–1236 cm^− 1^ (C–H bending and C–N stretching) and 1143–1023 cm^− 1^ (C–O stretching). The band at 895 cm^− 1^ is characteristic of β-type glycosidic linkages (Fig. 5a).

Principal Component 1 (PC1) accounted for 97.1% of the total variance and separated the samples along the X-axis in the PCA plot (Fig. 5b). Loading analysis indicated that the spectral region between 1150 and 950 cm^− 1^ contributed most to the separation of sample.

### Characterization of the protein extracts

#### Microstructure

**Fig. 6:**
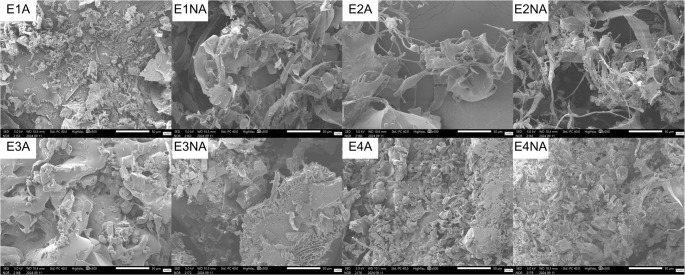
Scanning electron micrographs of protein extracts obtained from the mycelial biomass of *Ganoderma multiplicatum* 1962 produced under different submerged fermentation conditions, highlighting changes in fragmentation patterns. Representative images of extracts obtained under agitated (A) and non-agitated (NA) conditions. E: experiment (1–4)

Microstructural analysis of the protein extracts revealed distinct morphological patterns among the different fermentation conditions. Condition E1A showed a highly fragmented morphology, consisting of irregular lamellar plates with serrated edges. Similar, although less fragmented, features were also observed in E4A and E4NA. In contrast, extracts from E1NA, E2A, and E2NA displayed larger, thin, and more dispersed lamellar-like structures with a relatively uniform appearance. A third microstructural pattern was observed for E3A and E3NA, characterized by compact and aggregated structures forming dense clusters or granules (Fig. 6).

#### Biochemical composition

**Fig. 7:**
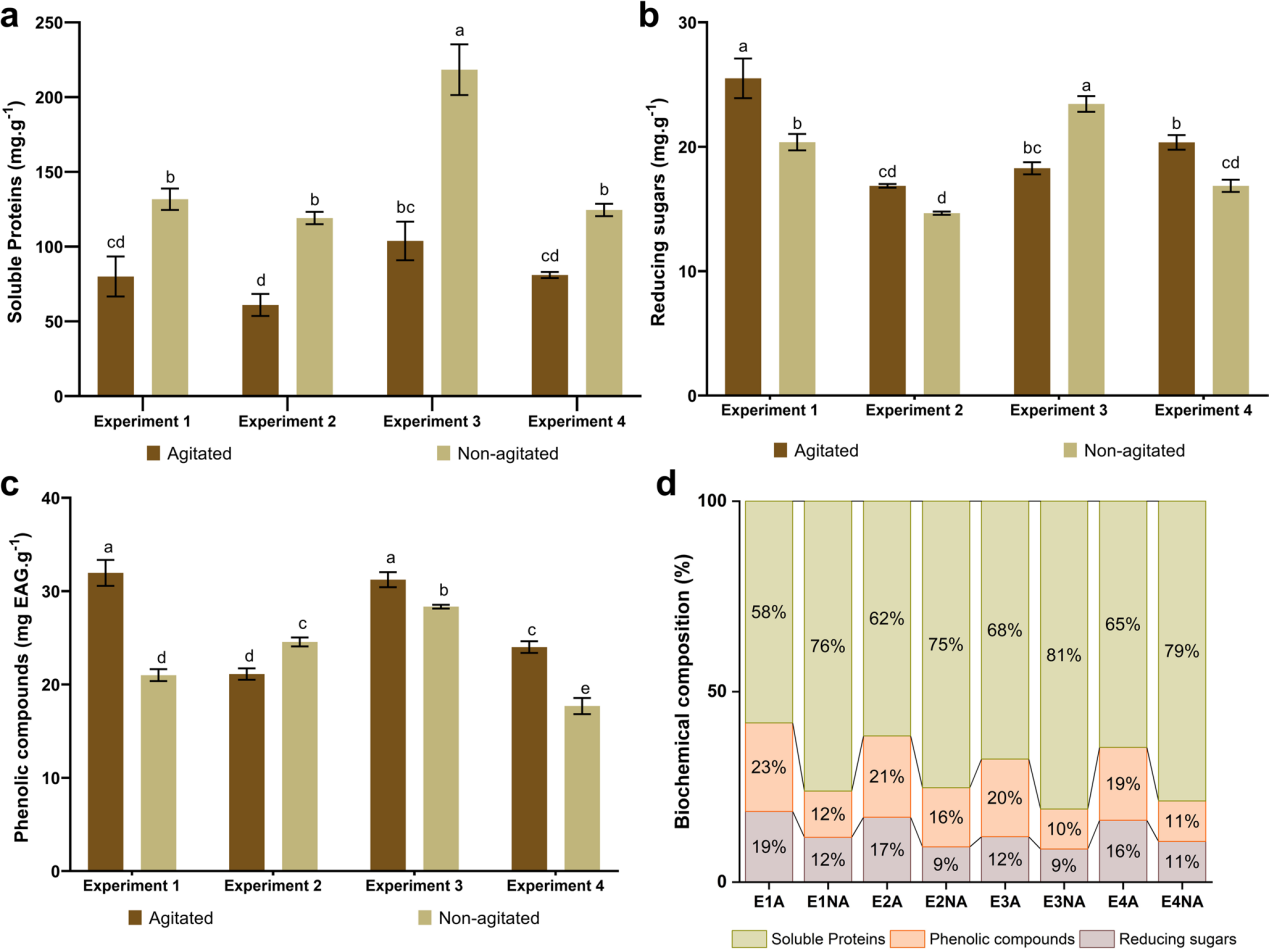
Concentration of (**a**) soluble proteins, (**b**) reducing sugars, (**c**) total phenolic compounds, and (**d**) the relative percentage composition of these biomolecules in *Ganoderma multiplicatum* 1962 protein extracts under different submerged fermentation conditions. Data are expressed as mean ± standard deviation (*n* = 3). Distinct letters above the bars indicate statistically significant differences between treatments (*p* < 0.05) according to one-way ANOVA followed by Tukey’s post hoc test

Soluble protein content was higher in extracts obtained under static conditions, particularly in E3NA, which reached 218.35 ± 16.95 mg g^− 1^ of extract (Fig. 7a). In contrast, higher levels of phenolic compounds and reducing sugars were observed under agitated conditions (Fig. 7b, c).

E3NA exhibited the highest protein percentage, whereas E1A showed the lowest (Fig. 7c). Although E1A had one of the lowest protein concentrations (80.03 ± 13.37 mg g^− 1^ of extract), it presented the highest values for reducing sugars (25.50 ± 1.59 mg g^− 1^ of extract) and total phenolic compounds (31.96 ± 1.39 mg GAE g^− 1^ of extract) (Fig. 7a–c). This pattern is also highlighted when considering the relative biochemical composition (Fig. 7d).

#### Antioxidant activity

**Fig. 8:**
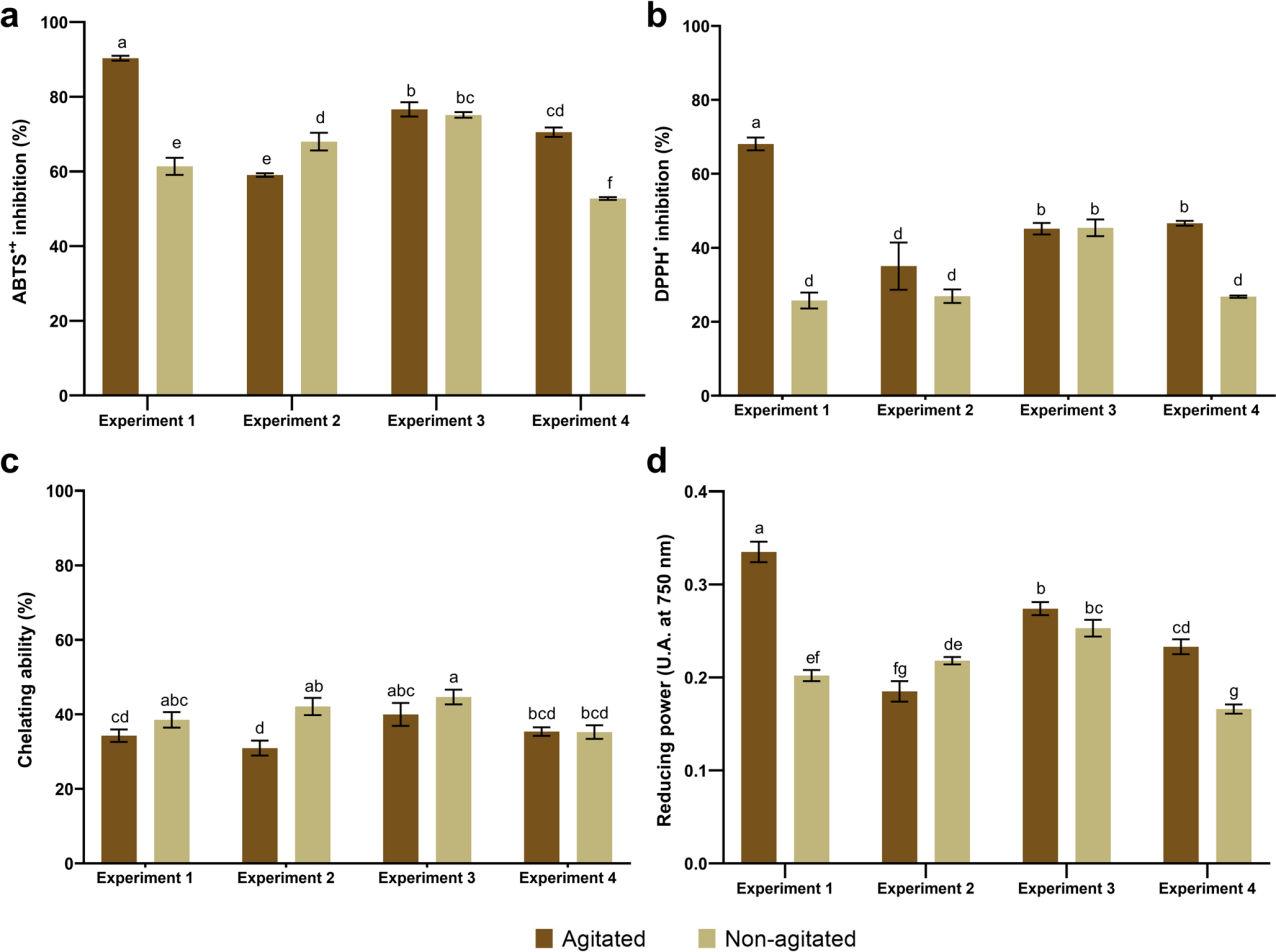
Antioxidant activity of *Ganoderma multiplicatum* 1962 protein extracts produced under different submerged fermentation conditions, evaluated by (**a**) ABTS^•+^, (**b**) DPPH^•^, (**c**) metal chelating ability, and (d) reducing power assays. Data are expressed as mean ± standard deviation (*n* = 3). Different letters above the bars indicate statistically significant differences between treatments (*p* < 0.05) according to one-way ANOVA followed by Tukey’s post hoc test

Extract E1A exhibited the highest radical scavenging activity, with inhibition values of 90.34 ± 0.66% (ABTS^•+^) and 68.09 ± 1.74% (DPPH^•^) (Fig. 8a, b). All samples showed low metal chelating activity, with E3NA presenting the highest value (44.66 ± 1.97%) (Fig. 8c). In the ferric reducing antioxidant power (FRAP) assay, E1A showed the highest absorbance (0.335 ± 0.011) (Fig. 8d).

**Fig. 9:**
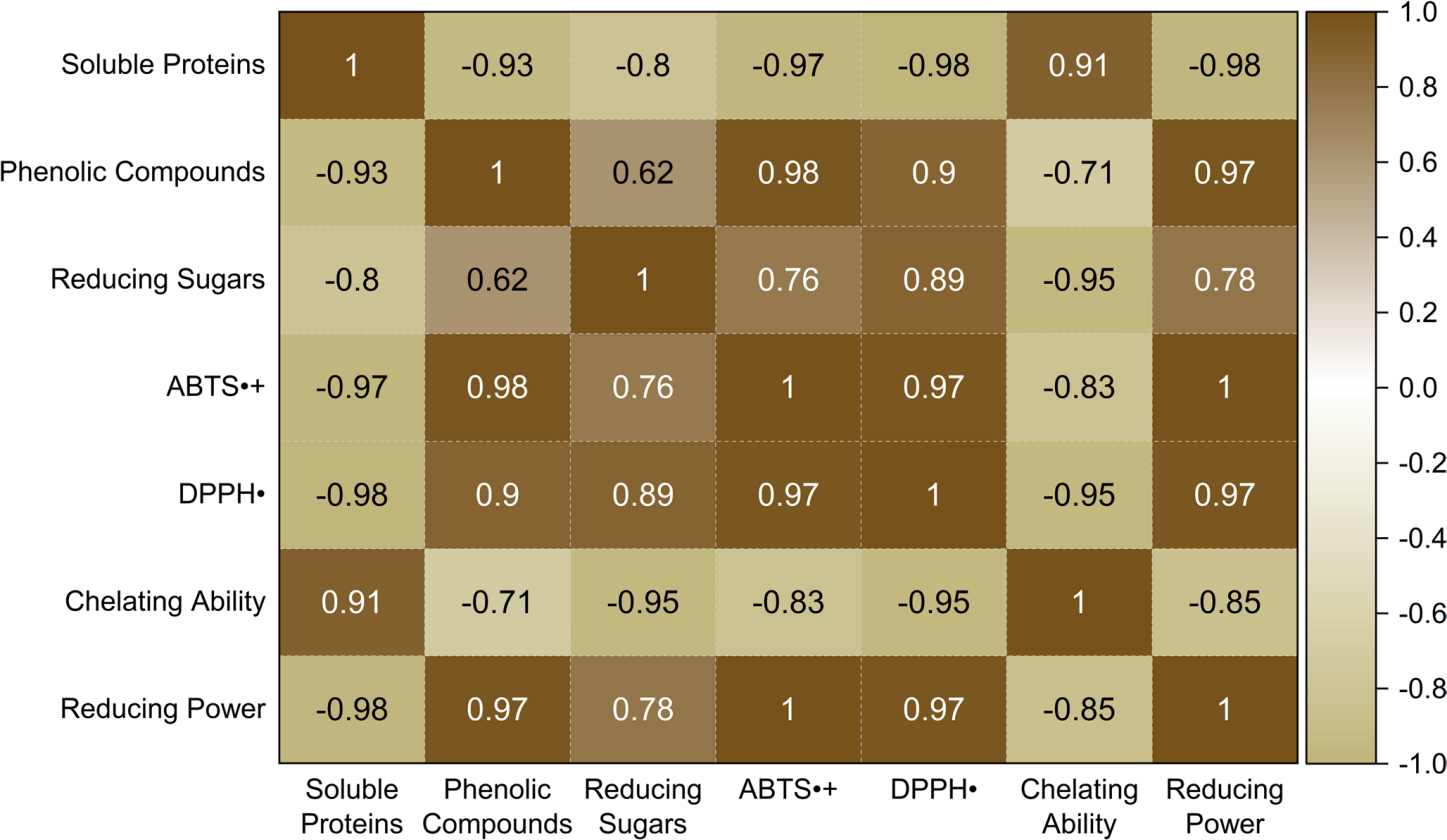
Pearson correlation matrix illustrating the relationships between soluble protein content, phenolic compounds, reducing sugars, and antioxidant activities (ABTS^•+^, DPPH^•^, metal chelating ability, and reducing power) under different submerged fermentation conditions. Color intensity represents the strength and direction of the Pearson correlation coefficients (r)

Pearson correlation analysis was performed to evaluate the relationships between protein content, total phenolic compounds, reducing sugars, and antioxidant activity (Fig. 9). Total phenolic compounds and reducing sugars showed positive correlations with ABTS^•+^, DPPH^•^, and reducing power. In contrast, metal chelating activity showed a positive correlation with soluble protein content.

## Discussion

Although a pure fungal culture had previously been obtained and provisionally identified as *Ganoderma* sp. 1962 based on macromorphological characteristics, molecular identification data for this isolate had not been reported to date [6]. Given the limitations of morphology-based identification within the genus *Ganoderma*, which exhibits high phenotypic plasticity, molecular markers are required for accurate species-level resolution. In this context, the internal transcribed spacer (ITS) region has been established as a universal DNA barcode for fungal identification [48]. The effectiveness of ITS sequencing for the authentication and identification of *Ganoderma* species and their commercial products is well documented, enabling reliable analysis across different sample types, including fresh mycelia and dehydrated materials [41, 49, 50].

*G*. *multiplicatum* is a neotropical species originally described in Brazil and is known for its morphological variability, which has led to historical misidentification as *G*. *lucidum* [51]. In Brazil, this species has been reported in several states, including Alagoas, Amazonas, Mato Grosso do Sul, Pará, Rio de Janeiro, Paraíba, Rondônia, and Roraima. Within the state of Amazonas, occurrences have been documented in the municipalities of Humaitá, Barcelos, and Manaus [52, 53].

Previous studies have reported the antimicrobial potential of this species against human pathogens, as well as antiproliferative effects on breast, lung, cervical, and colon tumor cell lines [54, 55]. However, these investigations have predominantly focused on secondary metabolites and polysaccharides derived from wild-collected basidiomata. Studies specifically investigating bioactive proteins from *G*. *multiplicatum* under submerged fermentation conditions remain limited.

The biomass values obtained in this study were higher than those reported by Silva et al. [8], who recorded a maximum production of 4.5 g L^− 1^ when cultivating the same strain under submerged fermentation for 14 days in Melin-Norkrans Modified (MNM) and Polysaccharide (POL) media. This enhanced biomass accumulation may be associated with differences in culture medium composition, particularly the use of readily assimilable organic nitrogen sources, such as yeast extract and soy peptone, which can reduce the metabolic cost of nitrogen assimilation by providing amino acids and peptides [56]. In addition, the absence of inorganic salt supplementation may have contributed to improved growth under the conditions evaluated, as similar trends have been reported for *Ganoderma curtissii* under submerged fermentation [57].

The composition of the culture medium appeared to be the main factor influencing biomass production, as no statistically significant differences were observed between agitated and static conditions within the same medium. Consistent with a previous study on *G*. *sichuanense* CC22 [9], higher biomass accumulation was observed at 20 g L^− 1^ glucose compared to 10 g L^− 1^, irrespective of soy peptone concentration. This trend may be related to differences in carbon-to-nitrogen ratios (C: N), with increased carbon availability supporting mycelial growth and biomass formation under the conditions evaluated.

Glucose is widely recognized as a key carbon source for mycelial growth and biomolecule production in *Ganoderma* [58] making it an important parameter to evaluate. By comparison, *G*. *sichuanense* CC22, a domesticated strain associated with stable growth and production performance, has been reported to exhibit glucose consumption limited to approximately 8 g L^− 1^ under submerged fermentation conditions [9]. In contrast, the wild Amazonian isolate evaluated here exhibited higher glucose consumption, suggesting a more dynamic carbon metabolism. This observation may reflect differences in metabolic behavior between wild and domesticated strains, highlighting the potential relevance of wild *Ganoderma* isolates for bioprocess development.

In submerged *Ganoderma* cultivation, glucose consumption ranging from 5 to 10 g L^− 1^ is generally considered high and has been associated with organic acid production, resulting in a concomitant decrease in pH [59], a pattern also observed in the present study, particularly under agitated conditions. Basidiomycetes cultivated under submerged conditions in the presence of glucose are known to produce organic acids such as oxalic acid and L-malic acid, as well as low-molecular-weight intermediates of the tricarboxylic acid cycle [60]. In addition, *Ganoderma* species can synthesize secondary metabolites collectively referred to as ganoderic acids, which may further contribute to medium acidification [61].

The E3NA treatment yielded the lowest biomass and was associated with reduced glucose uptake and minor pH variations compared to the other conditions. Similar findings have been reported for *G*. *sichuanense* CC22, in which lower biomass yields under static cultivation were associated with limited nutrient distribution due to the absence of mixing and homogenization mechanisms [9]. In this context, the C: N is a well-established factor influencing mycelial growth and metabolite production in cultivated mushrooms [62].

Medium composition and agitation are known to influence pellet formation, fungal morphology, and target product synthesis during submerged fermentation [63]. The observed variation in pellet size was associated with changes in the physicochemical properties of the culture broth as a function of medium composition, as described in previous studies [64]. Variations in medium coloration may be related to pigment synthesis, as reported for *G*. *lucidum* cultivated in the presence of peptone [65], or to the enzymatic degradation of medium components [66].

The adhesion of mycelial clusters to flask walls may negatively affect mycelial biomass production. This phenomenon has been described to occur when hyphae detach from pellets and, due to centrifugal and shear forces, are unable to re-establish pelleted growth, subsequently adhering to the glass surface [67]. Similar observations have been reported during the submerged fermentation of *G*. *lucidum*, where hyphal fragments exhibit a gelatinous, carbohydrate-rich layer at the glass–mycelium interface, a feature commonly associated with exopolysaccharide production [67, 68].

The micromorphological variations in hyphae observed between agitated and static cultures are consistent with previous descriptions for the genus *Ganoderma* [69–71]. The occurrence of staghorn-like hyphae under static cultivation, previously reported for *G*. *sichuanense*, has been associated with physiological responses to oxygen limitation. Under non-agitated conditions, diffusion-limited oxygen transfer may lead to localized hypoxic microenvironments, which can promote increased hyphal branching. Such morphological differentiation may increase the surface area in contact with the medium and facilitate mycelial expansion toward the air–liquid interface, potentially favoring growth under low-oxygen conditions and triggering adaptive metabolic responses, including increased intracellular protein accumulation [72, 73].

The ornamented spherical structures identified in this study have been previously described in *G*. *sichuanense* and may be associated with the storage of biomolecules or nutrients, possibly arising from hyphal vacuolization [74, 75]. The smooth spherical structures associated with hyphae (cell cuticles), as well as the observed clamp connections, correspond to characteristic morphological features already reported in submerged cultures of *Ganoderma* specie [69, 75–77].

The presence of chlamydospores may indicate an adaptive response to adverse conditions and has been associated with fast-growing phenotypes exhibiting thermotolerance [78, 79], which may be consistent with the production patterns observed in this study. The spiderweb-like structures correspond to the extracellular matrix (ECM) described in the submerged cultivation of other basidiomycetes. The ECM is composed of protein, carbohydrate, and lipid polymers that can facilitate adhesion, protection, and nutrient storage [80, 81].

Variations in FTIR-ATR absorbance profiles suggest that specific cultivation conditions may have influenced the accumulation of intracellular biomolecules, independently of overall biomass yield. Comparable spectral features have been described for basidiomata of other *Ganoderma* species and are generally associated with proteins, polysaccharides (β-glucans and chitin), and terpenoid compounds, including ganoderic and lucidenic acids [10]. The spectral region between 1150 and 950 cm^− 1^, which contributed most to the chemical differentiation among samples, is consistent with bands attributed to carbohydrate- and terpenoid-related structures, including those reported for *Ganoderma* secondary metabolites. These signals are commonly associated with C–O stretching, C–CH_3_ vibrations, and ring deformations [82].

Scanning electron microscopy (SEM) of the protein extracts (Fig. 6) revealed a highly fragmented microstructure in treatment E1A, suggesting the presence of smaller particle assemblies, which are known to influence the functional properties of protein materials [83]. Particle size is an important factor affecting protein solubility, as smaller particles generally exhibit a larger surface area available for hydration and solvent interaction [84]. Although average particle size was not directly quantified in this study, the microstructural characteristics observed for E1A suggest that this condition may favor the generation of protein extracts with improved solubility and dispersion. These aspects are relevant from an application perspective, as particle size and microstructural organization have been associated with techno-functional and biofunctional properties of protein extracts, influencing their potential for industrial applications [85, 86].

Treatment E3NA yielded the highest soluble protein content despite exhibiting the lowest fungal growth. In contrast, a previous study with *G*. *sichuanense* reported that a condition comparable to E3NA was associated with reduced biomass and protein production, likely due to a C: N ratio close to 1:1 [9]. This comparison suggests that protein accumulation under low C: N ratios may involve distinct metabolic responses among *Ganoderma* species. Nitrogen-rich conditions can increase the energetic demand for nitrogen assimilation and amino acid biosynthesis and have been reported to limit fungal growth while favoring protein synthesis. Accordingly, culture media based on organic nitrogen sources, which typically present low C: N ratios, have been described as favorable for protein accumulation [56, 87].

The higher protein recovery observed under non-agitated conditions is consistent with previous findings. When similar culture media and growth conditions were evaluated for *G*. *sichuanense*, a comparable trend toward increased protein recovery in biomasses from static cultures was reported, with values reaching approximately 400 mg g^− 1^ of extract [9]. This observation has been associated with the absence of shear forces, which can promote cell damage and leakage of intracellular contents under agitated conditions [72].

Protein quantification in this study was intended primarily for comparative analysis among fermentation conditions. However, direct comparison with absolute protein values reported in other studies should be made with caution, as protein–metabolite complex formation may interfere with colorimetric assays such as the Bradford method. This assay relies on the interaction of Coomassie Brilliant Blue with basic and aromatic amino acid residues; however, the binding of proteins to phenolic compounds or glycosylated moieties may partially mask dye-binding sites, leading to an underestimation of protein content [88, 89].

No consistent relationship was observed between soluble protein content and FTIR absorbance, which may be attributed to the contribution of insoluble components present in the mycelial biomass [90]. In *Ganoderma* species, polysaccharides and terpenoids are among the major constituents and can substantially contribute to infrared spectra, potentially masking variations associated with extractable proteins, phenolic compounds, and carbohydrates [91].

Higher soluble protein accumulation appeared to be associated with the short staghorn hyphae observed under static conditions (Fig. 4b, d, f, h). This hyphal morphology has been described in association with aerial mycelium development, which represents a growth strategy aimed at improving access to nutrients, including oxygen [69, 92]. Increased protein accumulation in mycelia cultivated under static conditions has also been reported for *G*. *sichuanense*, in which such differentiated hyphal forms have been interpreted as adaptive responses to environmental conditions that may directly or indirectly modulate biomolecule synthesis [72].

The characterization of the recovered proteins may be challenging due to their tendency to co-precipitate with low-molecular-weight compounds, such as phenolic compounds and carbohydrates [93]. Although dialysis is expected to remove these substances, this process may be ineffective when they are complexed with proteins [94], a scenario that may have occurred in the present study. However, the formation of protein–phenol and/or protein–carbohydrate complexes can modify protein properties and may enhance their biological activities, including antioxidant activity [95, 96].

Phenolic compounds may function as a chemical shield, limiting protein oxidation and enhancing conformational stability through non-covalent interactions [97]. Concurrently, carbohydrates are known to stabilize proteins by maintaining hydration layers and providing protection against physicochemical stresses such as dehydration, freezing, and thermal denaturation, mechanisms widely exploited in pharmaceutical and biotechnological formulations [98]. In this context, the higher concentration of phenolic compounds and carbohydrates in the E1A extract may favor the formation of stabilizing molecular assemblies that could preserve protein integrity and enhance functional performance.

The fragmented organization observed for the protein extract obtained under the E1A condition (Fig. 6) may be related to the higher contents of total phenolic compounds (TPC) and carbohydrates, which may interfere with protein–protein aggregation and hinder the formation of compact structures. This observation is consistent with previous reports on protein extracts of *G*. *sichuanense*, in which fragmented and fibrillar structures have been associated with higher phenolic content [72].

The antioxidant activity profiles observed in this study suggest that protein concentration alone does not account for the antioxidant potential of *G*. *multiplicatum* protein extracts. The superior antioxidant performance of the E1A extract may be associated with its distinct microstructural organization, as revealed by SEM, together with the higher content of total phenolic compounds (TPC) and reducing sugars, which may act synergistically to enhance bioactivity. In a previous study, Espinosa-García et al. [54] reported low antioxidant potential in organic extracts of *Ganoderma* cultivated under two-stage submerged fermentation, which was associated with low TPC content (13 mg GAE g^− 1^). These findings suggest that differences in cultivation conditions may influence the accumulation of antioxidant-related compounds.

Gu et al. [99] also reported higher antioxidant activity of *G*. *lucidum* peptides when assessed by the ABTS^•+^ assay compared to DPPH^•^, supporting the suitability of ABTS^•+^ for evaluating predominantly hydrophilic compounds. The low chelating activity observed in this study may be related to the reduced availability of functional groups such as hydroxyl (–OH), thiol (–SH), and carboxyl (–COOH), which are involved in metal ion complexation [100]. The reducing power values obtained are comparable to those reported for *G*. *lucidum*, in which absorbance values around 0.3 were observed at 20 mg mL^− 1^ for aqueous spore extracts [101] and polysaccharide fractions [102].

The observed correlations suggest that antioxidant activity in the protein extracts may arise from biochemical associations rather than from individual compounds acting alone. Redox-based assays were strongly associated with phenolic compounds and reducing sugars, supporting the contribution of protein–phenol and protein–carbohydrate interactions to antioxidant performance. In contrast, the positive correlation between protein content and metal chelating activity highlights a potential role of proteins in non-redox antioxidant mechanisms. These findings support a multifactorial model for antioxidant activity in the extracts.

Within the genus *Ganoderma*, several protein-based compounds have been reported to exhibit antioxidant activity, including selenium-enriched proteins, antioxidant enzymes, proteoglycans, glycopeptides, and protein extracts, highlighting their potential for pharmaceutical applications [103–107]. Consequently, the recovery of protein fractions with pronounced antioxidant activity, as observed in the present study, represents a promising strategy for adding value to *Ganoderma*-derived products, particularly those from underexplored Amazonian isolates. Notably, this study provides evidence of antioxidant protein potential in *G*. *multiplicatum* originating from the Brazilian Amazon.

## Conclusion

The fungal isolate 1962 was identified as *Ganoderma multiplicatum*, and its biomass composition and micromorphology were strongly modulated by submerged cultivation conditions. Static cultures promoted distinct hyphal architectures, including staghorn-like structures, which were consistently associated with higher soluble protein recovery. In contrast, cultivation in a medium containing 10 g L^− 1^ glucose, 5 g L^− 1^ yeast extract, and 2.5 g L^− 1^ soy peptone under agitated conditions resulted in protein extracts exhibiting superior antioxidant performance relative to the other conditions evaluated. This outcome is attributed to the concomitant recovery of proteins, phenolic compounds, and carbohydrates, favoring the formation of protein–metabolite complexes with enhanced functional activity. These findings indicate that agitation-driven submerged cultivation represents a viable strategy for obtaining antioxidant protein-rich extracts from Amazonian *G. multiplicatum*.

## Supplementary Information

Below is the link to the electronic supplementary material.

Supplementary Material 1

## Data Availability

All data generated during this study are included in this published article and its supplementary information files. Additional material is available from the corresponding author on reasonable request.
