# Supplementary material for: An Amazonian Ganoderma isolate as a source of antioxidant proteins: Influence of submerged fermentation conditions on mycelial architecture and bioactivity
Source: Braz J Microbiol. 2026 Apr 22;57(1):120. doi: 10.1007/s42770-026-01934-8 (PMC13103078; doi:10.1007/s42770-026-01934-8)
Supplement: Supplementary file 1 — Supplementary Material 1 [file 42770_2026_1934_MOESM1_ESM.pdf]

Supplementary Material

Annex 1. Information on the sequences used for the construction of the phylogenetic tree.

Species	Strain Code	Geographical Origin	NCBI Accession No.	Reference
Ganoderma sp.	1962	Amazonas, Brazil	PX736081	Present study
G. sichuanense	CC22	Asia	OQ691631	Author's data
G. acaciicola	Cui 16815 (type)	Australia	NR182900.1	[14]
G. alpinum	Cui 17467 (type)	Yunnan, China	NR182901.1	[14]
G. angustisporum	Cui 13817 (holotype)	Fujian, China	MG279170.1	[15]
G. applanatum	SFC20141001–24	South Korea	KY364255.1	[16]
G. aridicola	Dai 12588 (holotype)	Durban, South Africa	KU572491.1	[17]
G. australe	URM 83325	Brazil	JQ514106.1	[18]
G. bubalinomarginatum	Dai 20075 (type)	Guangxi, China	NR182905.1	[14]
G. carocalcareus	DMC 322 (holotype)	Cameroon, Africa	EU089969.1	[19]
G. castaneum	Cui 17283 (type)	Hainan, China	NR182903.1	[14]
G. casuarinicola	Dai 16336 (holotype)	Guangdong, China	MG279173.1	[17]
G. chalceum	URM 80457	Pernambuco, Brazil	JX310812	[20]
G. chocoense	QCAM 3123 (type)	Ecuador	MH890527	[21]
G. cocoicola	Cui 16791 (type)	Australia	NR182910.1	[14]
G. destructans	CBS 139793 (type)	Pretoria, South Africa	NR132919.1	[22]
G. dianzhongense	L4331 (type)	Yunnan, China	NR177159.1	[23]
G. eickeri	CMW49692 (type)	South Africa	MH571690	[24]
G. ellipsoideum	GACP14080966 (holotype)	Hainan, China	MH106867.1	[25]
G. enigmaticum	CBS 139792 (type)	Pretoria, South Africa	NR132918.1	[22]
G. guangxiense	Cui 14453 (type)	Guangxi, China	NR182906.1	[14]
G. hochiminhense	MFLU 19-2224 (holotype)	Vietnam	MN398324.1	[26]
G. knysnamense	CMW47755 (type)	South Africa	MH571681	[24]
G. leucocontextum	GDGM 40200 (holotype)	Tibet, China	KF011548	[27]
G. lingzhi	Wu 1006-38 (holotype)	Hubei, China	JQ781858.1	[28]
G. lucidum	MT26/10	Czech Republic	KJ143912.1	[29]
G. lucidum	Rivoire 4195	France	KJ143909.1	[29]
G. martinicense	LIP SWMart08-55 (type)	Martinique, France	KF963256.1	[30]

Species	Strain Code	Geographical Origin	NCBI Accession No.	Reference
G. meredithae	CBS 271.88	Louisiana, USA	NR164435.1	[31]
G. mizoramense	UMN-MZ4 (holotype)	India	KY643750.1	[32]
G. mbrekobenum	MIN 850481	Ghana, Africa	NR147647.1	[33]
G. multiplicatum	URM 83346	Paraíba, Brazil	JX310823	[20]
G. myanmarense	MFLU19-2167 (holotype)	Myanmar	MN396330.1	[26]
G. nasalanense	GACP17060211 (holotype)	Laos	MK345441.1	[34]
G. neojaponicum	AS5.541	Taiwan	AY593866.1	[35]
G. aff. oerstedii	URM 83400	Pernambuco, Brazil	JX310824	[20]
G. orbiforme	URM 83332	Pernambuco, Brazil	JX310813	[20]
G. oregonense	GO	Washington, USA	MT196417	[36]
G. ovisporum	HKAS123193	China	NR182704.1	[37]
G. parvulum	URM 83339	Pernambuco, Brazil	JX310817	[20]
G. podocarpense	QCAM6422 (holotype)	Ecuador	MF796661.1	[32]
G. resinaceum	MUCL52253	France	MK554786.1	[38]
G. ryvardenii	HKAS 58053 (type)	Cameroon, Africa	HM138671.1	[39]
G. sanduense	GACP18012501 (holotype)	China	MK345450.1	[34]
G. shanxiense	BJTC FM423 (holotype)	China	MK764268	[40]
G. sessile	111TX	USA	MG654306.1	[41]
G. sichuanense	HMAS 42798 (holotype)	Sichuan, China	JQ781877.1	[28]
G. sinense	Wei 5327	Hainan, China	KF494998.1	[15]
G. subangustisporum	Cui 18592 (type)	Yunnan, China	NR182909.1	[14]
G. subflexipes	Cui 17257 (type)	Guangdong, China	NR182904.1	[14]
G. sublobatum	Cui 16804 (type)	Australia	NR182907.1	[14]
G. thailandicum	HKAS104640 (holotype)	Thailand	MK848681.1	[26]
G. tongshanense	Cui 17168 (type)	Hubei, China	NR182908.1	[14]
G. tropicum	KUMCC 18–0046	Chiang Rai, Thailand	MH823539.1	[26]
G. tropicum	BCRC 37122	Taiwan, China	EU021457.1	[42]
G. weberianum	CBS128581	Taiwan	MK603805.1	[38]
G. weixiensis	HKAS100649 (holotype)	Yunnan, China	NR166271.1	[43]
G. wiiroense	MIN 938704 (type)	Ghana, Africa	NR158480.1	[44]
G. yunlingense	Cui 16288 (type)	Yunnan, China	NR182902.1	[14]

Species	Strain Code	Geographical Origin	NCBI Accession No.	Reference
Tomophagus colossus	TC-02	Vietnam	KJ143923.1	[29]
Tomophagus colossus	URM 83330	Paraíba, Brazil	JQ618247	[20]
